# Green-Making Stage Recognition of Tieguanyin Tea Based on Improved MobileNet V3

**DOI:** 10.3390/s26020511

**Published:** 2026-01-12

**Authors:** Yuyan Huang, Shengwei Xia, Wei Chen, Jian Zhao, Yu Zhou, Yongkuai Chen

**Affiliations:** 1 Institute of Digital Agriculture, Fujian Academy of Agricultural Sciences, Fuzhou 350003, China; 18759871027@163.com (Y.H.);; 2 School of Electronic, Electrical Engineering and Physics, Fujian University of Technology, Fuzhou 350118, China

**Keywords:** Tieguanyin tea, green-making stage recognition, MobileNet V3, Improved Coordinate Attention

## Abstract

The green-making stage is crucial for forming the distinctive aroma and flavor of Tieguanyin tea. Current green-making stage recognition relies on tea makers’ sensory experience, which is labor-intensive and time-consuming. To address these issues, this paper proposes a lightweight automatic recognition model named T-GSR for the accurate and objective identification of Tieguanyin tea green-making stages. First, an extensive set of Tieguanyin tea images at different green-making stages was collected. Subsequently, preprocessing techniques, i.e., multi-color-space fusion and morphological filtering, were applied to enhance the representation of target tea features. Furthermore, three targeted improvements were implemented based on the MobileNet V3 backbone network: (1) an adaptive residual branch was introduced to strengthen feature propagation; (2) the Rectified Linear Unit (ReLU) activation function was replaced with the Gaussian Error Linear Unit (GELU) to improve gradient propagation efficiency; and (3) an Improved Coordinate Attention (ICA) mechanism was adopted to replace the original Squeeze-and-Excitation (SE) module, enabling more accurate capture of complex tea features. Experimental results demonstrate that the T-GSR model outperforms the original MobileNet V3 in both classification performance and model complexity, achieving a recognition accuracy of 93.38%, an F1-score of 93.33%, with only 3.025 M parameters and 0.242 G FLOPs. The proposed model offers an effective solution for the intelligent recognition of Tieguanyin tea green-making stages, facilitating online monitoring and supporting automated tea production.

## 1. Introduction

China is the world’s largest producer and consumer of tea. In 2024, the total tea plantation area in China reached 3.50 million hectares, with a production of 3.50 million tons of dry tea and a total output value of 321.78 billion yuan [1,2]. The tea industry has become a vital pillar of rural revitalization. As a unique and high-value variety among Chinese teas, oolong tea enjoys worldwide renown [3]. Tieguanyin tea, as a typical representative of Oolong tea, has become one of the most widely consumed Oolong teas due to its unique flavor and aroma [4]. The unique quality of Tieguanyin, which “remains fragrant after seven infusions,” stems from its meticulous primary processing. The primary processing of Tieguanyin involves key steps, including sun-withering, green-making (also referred to as ZuoQing, fermentation, or oxidation), fixation, rolling, and drying. Among these, green-making is the critical stage for developing its characteristic aroma and flavor [5,6]. The green-making process comprises three to four alternating cycles of shaking (Yaoqing) and cooling (Liangqing). Shaking employs mechanical force to rupture leaf edge cells, promoting the oxidation of tea polyphenols, while cooling accelerates the conversion of internal compounds like amino acids and aromatic substances by evaporating moisture through spreading [7,8]. The degree of green-making directly determines the quality of the tea: insufficient green-making results in a weak aroma, while excessive green-making easily leads to “red stems and red leaves,” impairing its quality. Therefore, accurate identification of the green-making stages is a key prerequisite for ensuring the quality stability of Tieguanyin. However, current identification of the green-making stage in Tieguanyin production primarily relies on the experience and sensory evaluation of tea masters. This approach suffers from strong subjectivity, a lack of standardized criteria, and low efficiency. Therefore, introducing intelligent methods for stable recognition of the green-making process is urgently needed.

With the wide application of deep learning technology in tea production, this technology has become a key tool for promoting the development of smart agriculture. In tea quality classification, Guo et al. incorporated the Convolutional Block Attention Module (CBAM) into a ResNet model, achieving classification accuracies of 95.05% for oolong tea and 99.13% for black tea [9]. Similarly, Guo et al. developed an improved Inception network through image quality optimization and transfer learning, which improved the classification accuracy of tea appearance quality to 95% [10]. For applications in disease monitoring and internal quality analysis, Li et al. enhanced the MobileNet V3 architecture by integrating a Coordinate Attention (CA) module, attaining 95.88% accuracy in identifying tea pests and diseases [11]. Yang et al. combined CNN with visible–near-infrared spectroscopy for rapid detection of catechins and caffeine in tea, with the best-performing model achieving a coefficient of determination (R^2^) of 0.93 [12]. These studies confirm the capability of CNNs to effectively capture spatial and hierarchical image features, showing excellent performance in tasks such as quality inspection and pest recognition. Nevertheless, research on deep learning-based recognition of the green-making stages in Tieguanyin tea, which is a complex semi-fermented variety, remains limited. There is thus a pressing need to develop specialized models that balance recognition accuracy with lightweight design to satisfy real-time detection requirements in production environments.

This paper proposes a recognition model for the green-making stage of Tieguanyin, termed T-GSR, based on an improved MobileNet V3 architecture. The model employs a lightweight convolutional neural network to automatically identify green-making stages, thereby helping ensure consistent tea quality. The main contributions of this work are as follows:

(1) Image preprocessing and feature enhancement: Multi-color space fusion and morphological filtering techniques are applied to tea images to accurately segment key features associated with the green-making stage, such as brown spots and red edges, providing a reliable input basis for stage recognition.
(2) Adaptive residual learning: An adaptive residual branch is incorporated into the Bneck module, enabling effective feature fusion even when the numbers of input and output channels differ, thus preventing the loss of characteristic information across different green-making stages.
(3) Smoother activation with Gaussian Error Linear Unit (GELU): The Rectified Linear Unit (ReLU) activation function is replaced with the GELU, leveraging its smooth nonlinearity to stabilize the gradient descent process.
(4) Enhanced attention mechanism: An Improved Coordinate Attention (ICA) mechanism is introduced to replace the original Squeeze-and-Excitation (SE) module, strengthening the model’s ability to capture local fine-grained features and perceive similar color characteristics.

## 2. Materials and Methods

### 2.1. Sample Selection and Experimental Design

RGB images of Tieguanyin tea leaves at various green-making stages were collected at the Binghuai Family Farm in Longjuan Township, Anxi County, Fujian Province, China, on 3–4 May 2024. The green-making process consisted of three alternating cycles of shaking and cooling. To investigate over-green-making, an additional one-hour standing period was applied after the third cooling cycle, thereby defining the over-green-making stage. The green-making stages were defined on-site by three experienced tea masters. The specific processing parameters were as follows: the first shaking lasted 3 min, followed by a 50 min cooling; the second shaking lasted 7 min, followed by a 3 h cooling; and the third shaking lasted 15 min, followed by a 7 h cooling. Image data were acquired at six critical time points during the process: before the first cooling (Stage 1), after the first cooling (Stage 2), after the second cooling (Stage 3), 3 h after the start of the third cooling (Stage 4), moderate green-making (Stage 5), and over-green-making (Stage 6).

**Figure 1:**
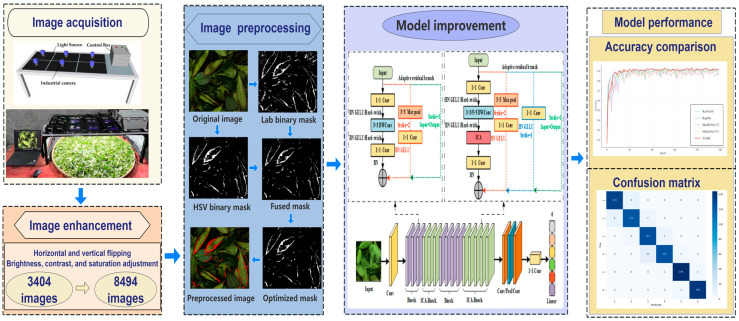
Experimental flowchart.

To enable stable imaging and efficient synchronous acquisition of tea leaf samples at six key processing stages, a custom six-channel RGB image acquisition system was developed. As illustrated in Figure 1 (image acquisition section), the system measures 120 cm × 48 cm × 35 cm and integrates six industrial RGB cameras (model: T-GE1000C-T-CL, HuaTengVision, Shenzhen, China; resolution: 3664 × 2748 pixels). Illumination was provided by six adjustable 20 cm × 20 cm flat-panel vision lights (model JH-FLK200200, Dongguan Juhua Vision Technology, Dongguan, China). To ensure lighting stability, the entire setup was enclosed with light-blocking fabric to minimize ambient interference. Using this system, a total of 3404 RGB images of Tieguanyin tea leaves across different green-making stages were obtained.

The workflow of this study is illustrated in Figure 1, which comprises four main phases: tea image acquisition and enhancement, image preprocessing, model improvement, and model evaluation. Model improvement constitutes the core focus of this research.

### 2.2. Image Data Acquisition

**Figure 2:**
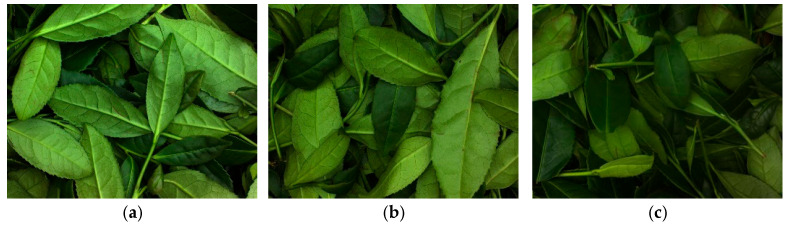

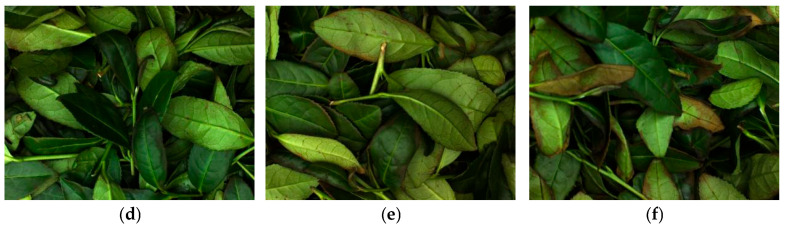
Images of different green-making stages: (**a**) Stage 1; (**b**) Stage 2; (**c**) Stage 3; (**d**) Stage 4; (**e**) Stage 5; (**f**) Stage 6.

Figure 2 shows the images of tea leaves captured at different green-making stages. As illustrated, the leaf color, brightness, and morphology differ significantly across these stages. Specifically, at Stage 1, the fresh leaves are vibrant and plump, with a rigid texture and glossy surface, exhibiting a bright emerald-green color and a strong vitality. At Stage 2, the leaves remain fresh and glossy; however, a slight drooping is observed compared to Stage 1. In Stage 3, a small number of brown spots emerge on the leaf petioles and surfaces, and the leaves begin to show a slight softening trend. By Stage 4, the brown spots on the petioles increase, and the brown areas along the leaf edges expand, forming slender strips. At Stage 5, the base of the petioles turns brown, and the leaf margins develop a distinct, evenly distributed “red edge” with an appropriate width, while the leaf texture becomes soft. At Stage 6, the “red-edged” area expands further, with some leaves even turning entirely tan. In summary, as the green-making process proceeds, the brown spots on the tea leaves gradually increase, the brown areas continue to expand, and the leaf morphology transitions from rigid to limp. These visual changes are attributed to the gradual decrease in moisture content and the enzymatic oxidation of polyphenols, leading to the formation of pigments such as theaflavins, thearubigins, and theabrownins [13].

### 2.3. Data Augmentation and Preprocessing

#### 2.3.1. Data Augmentation

The recognition of Tieguanyin tea green-making stages is a supervised learning task that requires a substantial number of annotated samples for model training. To improve the model’s generalization capability and mitigate overfitting, a series of data augmentation techniques were employed to generate additional training samples. These techniques included horizontal flipping, vertical flipping, and random adjustments to brightness, contrast, and saturation, thereby enhancing the diversity of the dataset.

After augmentation, the dataset comprised a total of 8494 images. The augmented dataset was partitioned into training, validation, and test sets in a 7:2:1 ratio. This data processing and partitioning pipeline remained consistent across all compared models. To mitigate the variability introduced by random splitting, each experiment was independently repeated five times. The final experimental result was reported as the average of the test outcomes from these five trials. The distribution of the augmented Tieguanyin green-making image dataset is summarized in Table 1.

#### 2.3.2. Image Preprocessing

**Figure 3:**
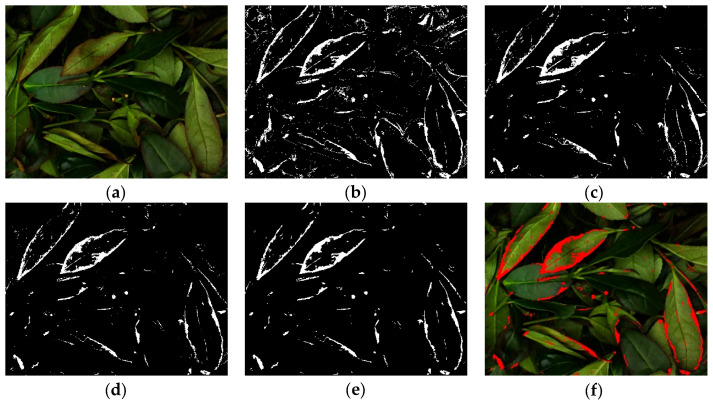
Images generated at each preprocessing step: (**a**) Original image; (**b**) Lab binary mask; (**c**) HSV binary mask; (**d**) Fused binary mask; (**e**) Optimized binary mask; (**f**) Preprocessed image.

A systematic image preprocessing workflow was designed to address the dynamic color changes in leaves and petioles during the green-making process of Tieguanyin tea. This workflow aims to automatically extract brown features associated with the green-making stages (e.g., brown spots on petioles and leaves) and replace them with red for enhanced feature distinctiveness, thereby reducing the complexity for subsequent model recognition. The preprocessing steps are detailed as follows:

(1) Color space conversion: The original RGB image (Figure 3a) was converted to the Lab color space using the OpenCV library to mitigate the influence of illumination and environmental variations and to better capture subtle color changes in brown regions during green-making. In the Lab color space, the L channel represents lightness, while the a and b channels represent color components from green to red and blue to yellow, respectively.
(2) Local contrast enhancement: Contrast Limited Adaptive Histogram Equalization (CLAHE) was applied separately to the a and b channels of the images. Since brown features on leaves and petioles may appear indistinct due to varying imaging conditions and uneven lighting, CLAHE effectively enhances the color contrast in target regions, providing clearer brown features for subsequent threshold segmentation and improving the accuracy of brown region extraction.
(3) Lab space thresholding: Fixed thresholds of 130 and 120 were applied to the enhanced a and b channels, respectively, for binarization. Pixels with intensity values exceeding these thresholds were set to 255 to preliminarily isolate potential brown areas, generating the Lab binary mask (Figure 3b). The determination of these thresholds was based on the empirical judgment of tea-making experts regarding the color changes in leaf stems and blades across different green-making stages, and their stability in segmentation performance was validated through repeated tests on multiple representative samples.
(4) HSV space thresholding: The original image was converted to the HSV color space. Threshold ranges were set as H ∈ [10, 30], S ∈ [50, 255], and V ∈ [50, 255] to extract potential brown regions, resulting in the HSV binary mask (Figure 3c). This threshold range was similarly refined under expert guidance and validated on diverse samples to ensure its robust adaptability across all green-making stages.
(5) Mask fusion: A pixel-wise logical AND operation was performed between the Lab and HSV binary masks to produce a fused binary mask (Figure 3d). This multi-color-space constraint mechanism enables precise segmentation of brown target regions in Tieguanyin green-making images.
(6) Morphological optimization and noise removal: The fused binary mask was refined using morphological closing and smoothing operations. An elliptical structuring element (5 × 5 pixels) was used for successive closing and opening operations to fill small holes inside targets and remove isolated noise points along the edges. Subsequently, external contours were extracted, and those with an area smaller than 100 pixels were filtered out, ensuring that the optimized binary mask (Figure 3e) retained only regions relevant to the browning features of petioles and leaves.
(7) Feature visualization: The optimized binary mask was used to replace the corresponding brown pixels in the original RGB image with red (RGB = [255, 0, 0]), yielding the final preprocessed image (Figure 3f). This step provides an intuitive visualization of local browning caused by enzymatic oxidation during the green-making process, establishing a reliable foundation for subsequent statistical analysis and classification.

**Figure 4:**
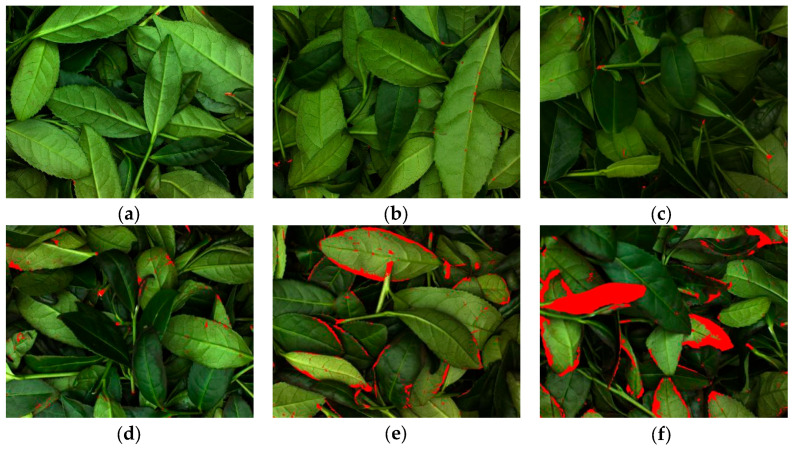
Preprocessed images of different green-making stages: (**a**) Stage 1; (**b**) Stage 2; (**c**) Stage 3; (**d**) Stage 4; (**e**) Stage 5; (**f**) Stage 6.

Images of different green-making stages after preprocessing are shown in Figure 4. Compared with Figure 2, it can be observed that the brown spots on petioles and veins, as well as the “red edge” features on leaf edges, are significantly enhanced in the images of each green-making stage. Thus, the proposed preprocessing step effectively improves feature representation and reduces the complexity for the subsequent model.

## 3. Tieguanyin Green-Making Stage Recognition Model

The T-GSR model (Tieguanyin Green-making Stage Recognition) proposed in this study was built upon the MobileNet V3-large [14] architecture. This foundation model was selected because it maintains a lightweight structure while offering powerful feature extraction capabilities, enabling it to effectively identify characteristic visual features of Tieguanyin tea leaves across different green-making stages, such as the “red-edged” appearance and the morphology of brown areas. Moreover, its high computational efficiency allows for potential deployment on mobile or embedded devices, thereby facilitating real-time intelligent management and broad application in the primary processing of Tieguanyin tea. The MobileNet V3 architecture has been widely adopted in tea image analysis tasks, such as tea disease identification [15], yellowing degree of yellow tea [16], and tea plant variety identification [17]. In these applications, it has demonstrated notable advantages by substantially reducing the number of parameters while maintaining high recognition accuracy.

**Figure 5:**
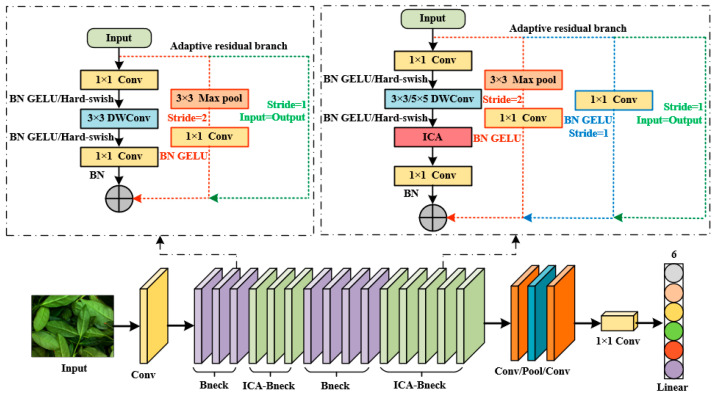
Architecture of the proposed T-GSR model.

Accurately distinguishing adjacent green-making stages remains challenging due to the high visual similarity between them and the significant color heterogeneity within a single tea leaf image. Furthermore, the original MobileNet V3 model was constrained by its inadequate capture of local discriminative features and suboptimal multi-level feature fusion, which collectively limited its recognition accuracy and stability [18,19]. To mitigate these limitations, this study introduced three key enhancements to the MobileNet V3 architecture. These improvements were designed to augment the model’s sensitivity to fine-grained characteristics of Tieguanyin tea during green-making, thereby boosting its generalization capability and classification accuracy while adhering to a lightweight design paradigm. The overall architecture of the proposed T-GSR model is depicted in Figure 5, with its comprehensive structural parameters provided in Table 2.

### 3.1. Introduction of the Adaptive Residual Branch

The residual structure facilitates the flow of information through neural networks by establishing shortcut connections, which not only mitigates the gradient vanishing problem but also accelerates model convergence. In the context of Tieguanyin green-making stage recognition, this structure aids in preserving and transmitting crucial visual features of tea leaves, enabling the model to learn from subtle feature variations more robustly and thereby enhancing final recognition accuracy.

**Figure 6:**
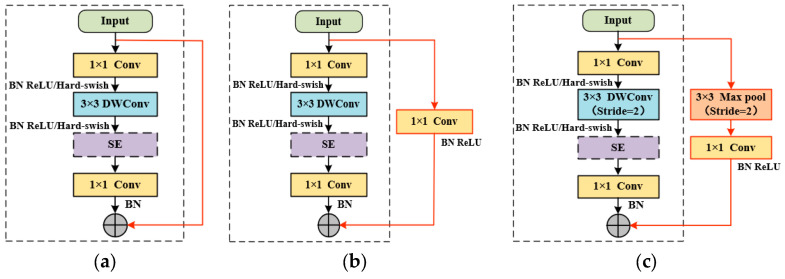
Diagrams of adaptive residual structures: (**a**) Stride = 1 and Input = Output; (**b**) Stride = 1 and Input ≠ Output; (**c**) Stride = 2.

In the original MobileNet V3 model, a residual connection is employed within a Bneck module only when the stride is 1 and the number of input channels matches that of the output channels, as depicted in Figure 6a. To enable effective feature fusion and minimize information loss even when the input and output channel dimensions differ, this study introduced an adaptive residual branch to the Bneck module.

As illustrated in Figure 6b, when the stride is 1 but the channel numbers are inconsistent, an additional residual branch composed of a standard 1 × 1 convolution is incorporated on the right side of the Bneck module. This branch functions to adjust the channel dimensions to align with those of the main branch, thereby achieving effective linear feature combination and channel alignment without altering the spatial size of the feature maps.

When the stride of the 3 × 3 depthwise convolution is 2, the spatial dimensions of the output feature map are reduced, preventing its direct summation with the input feature map. To address this issue, a specialized residual branch is designed on the right side of Figure 6c to connect the two. This branch comprises a max-pooling layer for downsampling and a 1 × 1 standard convolution for feature extraction, ensuring the preservation of essential semantic information after spatial down-sampling.

It should be noted that the dashed boxes in Figure 6 represent the original Bneck structures, in which the SE attention module is applied only to specific Bneck blocks. The detailed configuration of SE usage is indicated by the “√” marks in Table 2.

### 3.2. Replacement of Activation Function

The original MobileNet V3 model utilizes the ReLU activation function in its first six Bneck modules. Although ReLU is widely adopted in various neural networks due to its computational efficiency and simplicity, and it effectively mitigates the vanishing gradient problem, its non-differentiability at zero may lead to information loss when discerning subtle features in Tieguanyin green-making images [20]. This limitation can impede precise gradient updates and ultimately constrain the model’s recognition performance.

**Figure 7:**
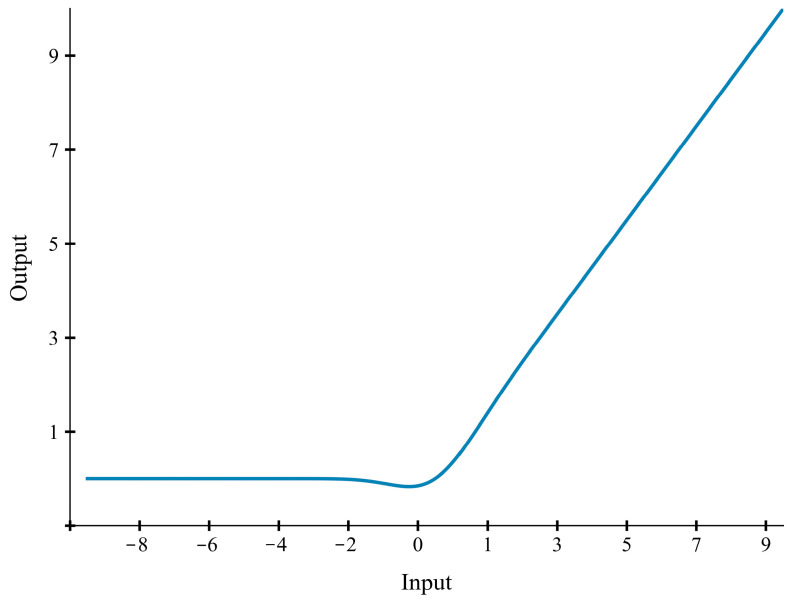
The GELU activation function.

In the proposed T-GSR model, the ReLU activation function was replaced with the GELU. As illustrated in Figure 7, the GELU activation function is characterized by its continuity and stochastic regularity, yielding a smoother output that stabilizes the gradient descent process [21,22]. This property enabled the model to more accurately capture fine-grained variations in leaf color and morphology during the Tieguanyin green-making process, thereby improving the overall recognition accuracy.

### 3.3. Improved Attention Mechanism

**Figure 8:**
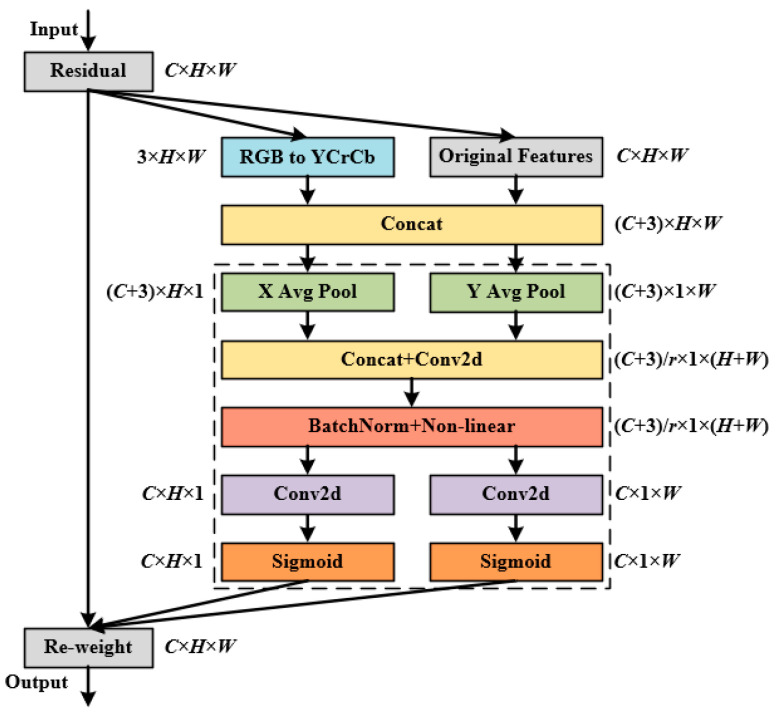
Structure of the ICA module.

The accurate identification of Tieguanyin green-making stages can be influenced by multiple factors, including leaf color, morphology, the quantity and distribution of brown spots, as well as ambient lighting and shadow interference. The SE attention mechanism in MobileNet V3 relies exclusively on global average pooling to extract global statistics across channels, which it then uses to recalibrate feature responses. This approach fails to incorporate spatial positional information, thereby limiting its ability to capture subtle local variations in images [23,24]. In contrast, the CA mechanism introduces two-dimensional coordinate information during the channel compression process, enabling collaborative modeling of spatial perception and channel-wise attention. By embedding positional encoding along both horizontal and vertical directions, CA not only preserves complete spatial information but also achieves effective integration across both channel and spatial dimensions. This allows for more precise detection of fine brown spots and their distribution patterns in tea leaf images [25]. Building upon the original CA framework, this study proposed an ICA mechanism. The ICA architecture incorporates key structural enhancements to better address the specific challenges in tea leaf feature extraction, as illustrated in Figure 8.

The workflow of the ICA module is as follows:

(1) Multi-color space feature fusion: The input feature map with dimensions C × H × W (in RGB color space) is transformed into the YCrCb color space. In this space, the Y channel (luminance) effectively highlights illumination variations in tea leaves, which helps capture variations in illumination and shading on the tea leaf surface, while the Cr and Cb channels more effectively represent chromatic features in leaf blades and stems. Then, the converted YCrCb features are concatenated with the original RGB features along the channel dimension, yielding a feature map of size (C + 3) × H × W. By introducing color space features that decouple luminance from chrominance, this approach preserves the original color information while providing richer and more discriminative color feature representations for subsequent attention modeling.
(2) Multi-directional global feature extraction: Average pooling is performed separately along the height (H) and width (W) dimensions of the (C + 3) × H × W feature map, yielding two feature maps of sizes (C + 3) × H × 1 (denoted as X-direction features) and (C + 3) × 1 × W (denoted as Y-direction features). This design enables the model to capture distinct global features along both spatial directions independently.
(3) Cross-directional feature fusion and dimensional adjustment: The X- and Y-direction features are first concatenated and then processed by a 1 × 1 convolution for channel reduction. This yields a feature map of dimensions (C + 3)/r × 1 × (H + W), where r denotes the channel reduction factor, which is set to 32 to balance the model’s representational capacity with computational efficiency. This operation achieves fusion of global features from different directions while helping the model focus on the spatial distribution of relevant features in green-making images. Subsequently, the fused features undergo batch normalization and nonlinear activation to enhance their representational capacity, producing an intermediate feature vector $f\;=\;R^{(C+3)/r\times 1\times \left(H+W\right)}$. Finally, this feature map f is split along the spatial dimension into two separate vectors: $f_{h}\;=\;R^{(C+3)/r\times H\times 1}$ and $f_{w}\;=\;R^{(C+3)/r\times 1\times W}$.
(4) Attention weight generation and feature enhancement: A 1 × 1 convolution is used to increase the channel dimensions f_h_ and f_w_, obtaining feature vectors $f_{h\_conv}=R^{C\times H\times 1}$ and $f_{w\_conv}=\;{\;R}^{C\times 1\times W}$. This ensures consistency in the number of channels between the output attention maps and the input feature map for subsequent feature fusion. Subsequently, the Sigmoid activation function is applied to generate attention weights $g_{h}\;=\;R^{C\times H\times 1}$ (corresponding to the height direction) and $g_{w}\;={\;R}^{C\times 1\times W}$ (corresponding to the width direction). Finally, the input C × H × W feature map is multiplied element-wise with g_h_ and g_w_, resulting in a feature map with enhanced feature representation at the module output.

## 4. Experimental Setup and Evaluation Metrics

### 4.1. Experimental Setup

The experiments were conducted on a computer running the Windows 11 operating system, equipped with an Intel Core i7-13700H CPU (Intel, Santa Clara, CA, USA) and an NVIDIA GeForce RTX 3090 GPU (NVIDIA, Santa Clara, CA, USA). The model was developed in the PyCharm 2023.2 platform using Python 3.9 and PyTorch 2.1.1 framework. For accelerated computation, the CUDA and cuDNN libraries were leveraged. The model was trained with the Adam optimizer and the cross-entropy loss function. The training configuration was as follows: batch size of 16, initial learning rate of 0.001, and a total of 100 epochs. The validation set was used to evaluate the model after each epoch, and the model weights along with the validation results were saved for further analysis.

### 4.2. Evaluation Metrics

To comprehensively evaluate model performance, we employed accuracy (Acc), precision (Pre), recall (Rec), and the F1-score as the primary metrics, while model complexity was quantified by the total number of parameters and floating-point operations (FLOPs) [26]. For the multi-class task of Tieguanyin green-making stage identification, the precision, recall, and F1-score for each class were calculated using the One-vs-the-Rest (OvR) strategy. In this approach, each class is sequentially treated as the positive class, with all other classes considered negative. The overall performance metrics were then computed via macro-averaging to ensure equal consideration of all stages and to prevent the evaluation from being skewed by class imbalance. The formulas for the four evaluation metrics are given below:

(1) $$\mathrm{Accuracy}\;=\;\frac{\mathrm{TP}\;+\;\mathrm{TN}}{\mathrm{TP}\;+\;\mathrm{TN}\;+\;\mathrm{FP}\;+\;\mathrm{FN}}$$

(2) $$\mathrm{Precision}=\frac{\mathrm{TP}}{\mathrm{TP}+\mathrm{FP}}$$

(3) $$\mathrm{Recall}=\frac{\mathrm{TP}}{\mathrm{TP}+\mathrm{FN}}$$

(4) $$\mathrm{F1-score}=2\;\times \;\frac{\mathrm{Pre}\;\times \;\mathrm{Rec}}{\mathrm{Pre}+\mathrm{Rec}}$$

where TP denotes true positives (correctly predicted positive samples); TN denotes true negatives (correctly predicted negative samples); FP denotes false positives (negative samples incorrectly predicted as positive); and FN denotes false negatives (positive samples incorrectly predicted as negative).

## 5. Experimental Results and Analysis

### 5.1. Impact of Adaptive Residual Branches on Model Training

**Figure 9:**
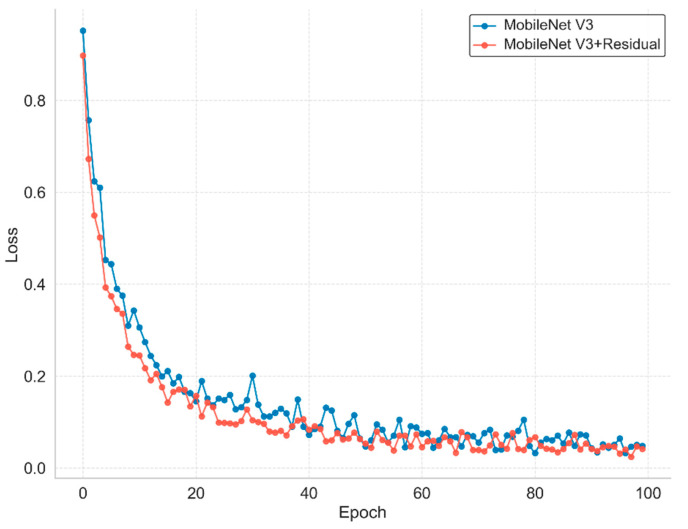
Comparison curves of model loss values.

Figure 9 compares the training loss curves of the model with and without the incorporation of adaptive residual branches. As shown, the model equipped with residual branches exhibits a steeper descent in loss during the initial training phase (approximately the first 40 epochs), signifying an accelerated convergence rate. Furthermore, compared to the baseline without residual connections, the loss curve of the enhanced model displays reduced oscillations in the mid-to-late training stages, indicating superior convergence stability. This smoother and more monotonic decline in loss suggests that the adaptive residual structure effectively mitigates the vanishing gradient problem, thereby providing a more stable optimization path and enhancing the model’s robustness in later training phases.

### 5.2. Impact of Different Activation Functions on Model Performance

The proposed T-GSR model introduced the GELU activation function to replace the original ReLU function in the MobileNet V3 architecture. As a smooth nonlinearity based on Gaussian distribution, GELU does not zero out negative inputs but preserves scaled negative values according to their magnitude. This property makes GELU more continuous and smooth compared to ReLU, thereby enhancing the model’s capacity for fine-grained feature representation. To systematically evaluate different strategies for handling negative inputs, we additionally incorporated the LeakyReLU activation function in our experiments. Unlike ReLU, LeakyReLU outputs αx for negative values, preserving a controlled amount of negative information [27]. Furthermore, to examine the effect of constraining positive outputs, we included ReLU6 in our comparative analysis. While similar to ReLU in zeroing negative inputs, ReLU6 imposes an upper limit of six on positive outputs [28]. The recognition performance of the model with these four distinct activation functions is comprehensively compared in Table 3.

As summarized in Table 3, the models with LeakyReLU and ReLU6 activations demonstrated modest but consistent improvements across all evaluation metrics for green-making stage recognition compared to the original ReLU-based model. Notably, the GELU-activated model achieved superior performance, reaching 92.32% accuracy and an F1-score of 92.28%, thereby outperforming all other activation functions tested. This superior performance confirmed that GELU’s combination of partial negative value preservation and smooth nonlinear transformation effectively enhances the model’s capacity to capture discriminative feature details.

### 5.3. Impact of Replacement with the ICA Mechanism on Model Performance

#### 5.3.1. Impact of ICA Mechanism Replacement on Green-Making Feature Recognition

To intuitively demonstrate the effectiveness of replacing the original SE attention mechanism with the proposed ICA mechanism, this study employed Gradient-weighted Class Activation Mapping (Grad-CAM) to visualize the model’s focus on Tieguanyin tea green-making features. Grad-CAM generates heatmaps that highlight the regions of interest, attention intensity, and distribution of discriminative features, thereby revealing the visual basis for the model’s classification decisions [29].

**Figure 10:**
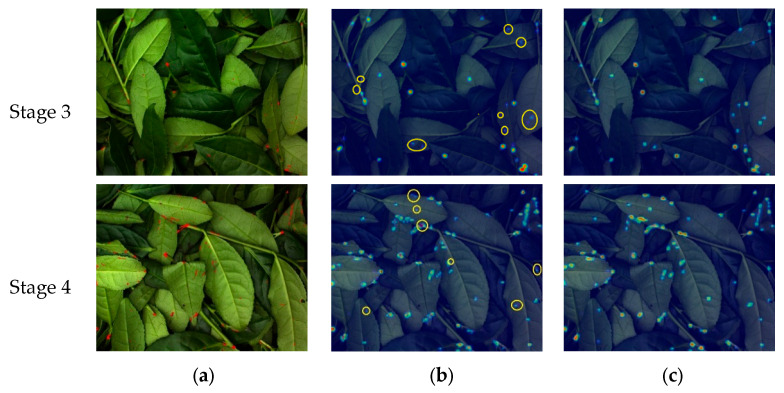
Comparison of feature localization capabilities before and after replacing the attention mechanism: (**a**) Preprocessed original images, (**b**) Heatmaps generated by the SE attention mechanism, (**c**) Heatmaps generated by the ICA mechanism.

A comparison of feature localization before and after replacing the attention mechanism is presented in Figure 10. The first and second rows display sample images from Stage 3 and Stage 4, respectively. As shown in Figure 10a, the preprocessed images of these two consecutive stages exhibit high similarity in leaf color, morphology, and the distribution of brown spots. The original SE attention mechanism in MobileNet V3 showed limited attention and failed to detect some fine brown spots (which appear as red spots after preprocessing), as highlighted by the yellow circles in Figure 10b. In contrast, as illustrated in Figure 10c, the incorporation of the ICA significantly strengthened the model’s focus on subtle red spots on petioles and leaf surfaces, with all relevant spots being accurately identified. This validated the effectiveness of integrating the ICA.

Therefore, by replacing the SE attention mechanism with ICA, the model’s sensitivity to subtle variations in color and morphology within Tieguanyin tea images was enhanced, allowing the improved model to more reliably capture key features associated with shaking and cooling during the green-making process.

#### 5.3.2. Impact of Different Attention Mechanisms on Model Performance

To evaluate the effectiveness of the proposed ICA mechanism in improving the recognition accuracy of the green-making process, we compared it against three established attention mechanisms—CBAM [30], ECA [31], and CA—by replacing the original SE module in the MobileNet V3 architecture with each of them. All models were trained and evaluated under the same experimental conditions. The corresponding results are summarized in Table 4.

According to the results in Table 4, the recognition model equipped with the CBAM attention mechanism failed to surpass the SE-based baseline model across all performance metrics. This suggests that CBAM exhibits relatively weak feature extraction capability for tea leaf images and struggles to adapt to the fine-grained feature distribution required in this task, particularly in distinguishing subtle variations during the green-making process.

In comparison, models incorporating the ECA and CA mechanisms demonstrated moderate yet consistent improvements, achieving accuracy rates of 92.79% and 93.03%, respectively, with corresponding F1-scores of 92.77% and 92.97%. These results indicate that both ECA and CA offer better adaptability to the feature patterns present in green-making images.

In contrast, ICA, which introduces YCrCb color space fusion, further improved model performance while maintaining a lightweight network architecture. This indicates that multi-color-space feature fusion enhances the model’s ability to capture subtle color transitions throughout the green-making stages. The ICA-equipped model achieved the highest scores across all evaluation metrics, reaching an accuracy of 93.38%. This further confirmed that ICA more effectively directs the model’s attention toward discriminative visual cues such as leaf browning, thereby improving both the accuracy and overall discriminative capacity of green-making stage identification.

### 5.4. Ablation Experiment Results of the T-GSR Model

To evaluate the effectiveness of the preprocessing techniques and the proposed architectural improvements, we performed ablation experiments; the results are summarized in Table 5.

As shown in Table 5, the sequential incorporation of image preprocessing, the adaptive residual branches, the GELU activation function, and the ICA mechanism progressively enhanced key performance metrics, including accuracy and F1-score.

A comparison between the first and second rows indicates that image preprocessing alone improved the recognition accuracy of the baseline MobileNet V3 model by 1.07%, confirming the value of preprocessing for enriching feature representation.

Controlled experiments were further conducted to verify the independent contribution of each improvement module. Building upon the model using only image preprocessing, the separate introduction of the GELU activation function (row 3) increased the model accuracy and F1-score to 91.96% and 91.91%, respectively. Meanwhile, the separate introduction of the ICA mechanism (row 4) further raised these two metrics to 92.08% and 92.07%. This indicates that both GELU and ICA can independently enhance the model’s ability to represent features of the green-making stages without relying on other structural modifications.

Finally, the complete T-GSR model (last row), which integrates all strategies, demonstrated significant advantages over the preprocessing-enhanced baseline (row 2): accuracy improved by 2.36% and the F1-score increased by 2.41%. Furthermore, while the computational cost remained largely unchanged, the number of model parameters was reduced by approximately 28.15%, achieving a higher degree of lightweight design. This facilitates deployment on terminal devices with limited hardware resources.

### 5.5. Performance Comparison Between T-GSR Model and Other Models

To comprehensively evaluate the performance of the T-GSR model, we carried out comparative experiments with several established models, including conventional architectures such as ResNet18 [32] and RegNet [33], as well as lightweight networks like ShuffleNet V2 [34] and MobileNet V3.

#### 5.5.1. Accuracy Comparison

**Figure 11:**
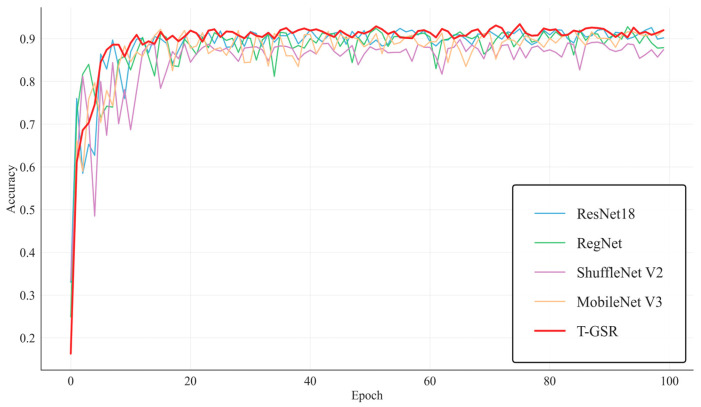
Comparison of Model Accuracy Curves.

Figure 11 shows the recognition accuracy curves of the different models on the Tieguanyin green-making validation set. In the first 10 epochs, the accuracy of all models rose rapidly, suggesting their effectiveness in learning shallow features from different green-making stages. Between 10 and 50 epochs, the accuracy of T-GSR continued to grow steadily from approximately 85% to nearly 90%, whereas the other models exhibited noticeably slower progress. The curves of RegNet, ShuffleNet V2, and MobileNet V3 displayed substantial oscillations, indicating certain instabilities during training. In contrast, the T-GSR curve was notably smoother, underscoring its stronger capability in feature fusion and deep semantic extraction. In the final validation phase (after epoch 50), the accuracy curve of T-GSR plateaued at over 91%, confirming that the model had converged without significant oscillations. Although the other models also converged to some extent, their overall accuracy remained lower than that of T-GSR, and their curves still exhibited visible fluctuations. The high final accuracy and low variance of T-GSR collectively demonstrate its excellent robustness and resistance to overfitting.

#### 5.5.2. Comprehensive Performance Comparison

To further evaluate model performance, comprehensive testing was conducted on the test dataset using four models: ResNet18, RegNet, ShuffleNet V2, and T-GSR. The results are presented in Table 6.

As shown in Table 6, among the conventional models, RegNet achieved superior performance over ResNet18 across all evaluated metrics. Compared to RegNet, the T-GSR model achieved a 2.07% higher accuracy, while utilizing 46.65% fewer parameters and requiring 60.71% fewer FLOPs. When compared to the lightweight ShuffleNet V2, T-GSR demonstrated substantial advantages, outperforming it by over 5% in both accuracy and F1-score, while also reducing both the parameter count and FLOPs by more than 40%. In summary, the T-GSR model delivers outstanding performance in both recognition accuracy and computational efficiency, underscoring its powerful capability to discern subtle color variations and complex features characteristic of the green-making process.

#### 5.5.3. Confusion Matrix Analysis

**Figure 12:**
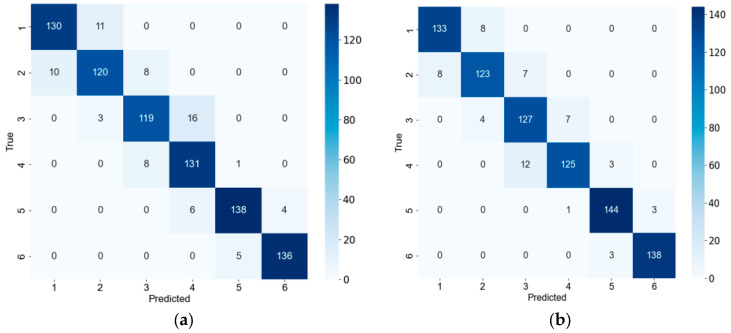
Confusion matrices of the test set: (**a**) RegNet model; (**b**) T-GSR model. Note: 1 represents Stage 1; 2 represents Stage 2; 3 represents Stage 3; 4 represents Stage 4; 5 represents Stage 5; 6 represents Stage 6.

The confusion matrix serves as a key tool for evaluating the classification performance of deep learning models, providing an intuitive numerical representation of a model’s correct and incorrect predictions. As indicated in Table 6, the RegNet model showed the strongest classification performance among the compared baseline models (ResNet18 and ShuffleNet V2). Figure 12 displays the confusion matrices for the RegNet and T-GSR models on the test set.

Figure 12 reveals that both models display some misclassification between adjacent green-making stages, reflecting the continuous nature of the Tieguanyin process where image features evolve gradually. Notably, stage 4 presents the most significant classification bottleneck. The reasons for this can be further analyzed with reference to Figure 2 (raw images) and Figure 4 (preprocessed images). From Figure 2, it can be seen that the leaves at stage 4 are in a transitional state between stages 3 and 5 in terms of reddening patterns along the leaf margins and overall coloration: localized red edges and browning begin to appear, but a continuous and stable morphological pattern has not yet formed. Further observation of the preprocessing results in Figure 4 reveals that the browning regions extracted at stage 4 are relatively small and scattered in distribution. Their spatial connectivity is noticeably weaker than in stage 5, yet more pronounced than in stage 3. This instability in the quantity, location, and continuity of such browning features causes stage 4 to overlap with adjacent stages in the feature space, thereby increasing the difficulty of model discrimination.

Despite this challenge, the T-GSR model outperforms RegNet across all other stages, committing 16 fewer errors in total. This demonstrates that the proposed T-GSR model possesses superior feature-modeling capability and overall classification performance for identifying Tieguanyin green-making stages.

## 6. Conclusions

This paper presented T-GSR, a lightweight model based on MobileNet V3, designed for accurate recognition of Tieguanyin tea green-making stages. Through a series of targeted improvements, we have validated its superiority in this specialized task.

First, we designed a multi-color space fusion and morphological preprocessing pipeline to accurately segment and visualize critical features, including brown spots and the distinctive “red-edged” characteristics on tea leaves and petioles, thereby providing more discriminative input for subsequent model training. Second, we introduced adaptive residual branches into the Bottleneck (Bneck) modules to mitigate information loss when input and output channel dimensions differ, which significantly accelerated gradient propagation and improved convergence. Third, we replaced the ReLU activation function with the continuously smooth GELU, resulting in a more stable training process with markedly reduced loss oscillations. Furthermore, the original SE mechanism was substituted with our ICA. By enabling spatial-channel collaborative modeling, ICA substantially enhanced the model’s sensitivity to subtle local features in tea leaves.

A dedicated dataset of 8494 images was constructed, capturing six key stages of the Tieguanyin green-making process, known as the “three shaking and three cooling” cycles. Extensive experiments conducted on this dataset confirmed the contribution of each proposed improvement. Ablation studies demonstrated that every optimization positively impacted the final performance. Compared to the original MobileNet V3, the complete T-GSR model achieved a significant overall performance gain, with accuracy increasing by 3.43% and the F1-score by 3.44%, while the number of parameters was reduced by nearly 30% without increasing computational overhead.

In comparative experiments against several commonly used models, including ResNet18, RegNet, ShuffleNet V2, and MobileNet V3, T-GSR consistently outperformed all others, achieving at least a 2% higher accuracy while reducing both the parameter count and FLOPs by over 40%.

In summary, T-GSR significantly enhances the accuracy and stability of Tieguanyin green-making stage recognition while maintaining minimal computational cost, demonstrating strong potential for deployment on mobile and embedded devices in production environments. It should be noted that, to ensure consistency in data preprocessing, this study applied data augmentation prior to dataset splitting. Future work will adopt a more rigorous splitting strategy to further enhance evaluation rigor. Moreover, the quantitative relationship between different green-making stages and the proportion of browning areas warrants further systematic investigation. Integrating explicit area statistics with deep representations could improve model interpretability. Subsequent efforts will focus on model quantization, hardware acceleration, and online inference optimization, ultimately supporting real-time, intelligent tea processing and quality monitoring.

## Figures and Tables

**Table 1:** Tieguanyin green-making image dataset.

Stage	Training Set	Validation Set	Test Set	Total
Stage 1	994	283	141	1418
Stage 2	970	276	138	1384
Stage 3	974	277	138	1389
Stage 4	987	281	140	1408
Stage 5	1039	296	148	1483
Stage 6	989	282	141	1412
Total	5953	1695	846	8494

**Table 2:** Architectural parameters of the T-GSR model.

Input	Operator	Output Channel	ICA Module	Activation Function	Stride
2242 × 3	Conv2d	16	-	Hard-swish	2
1122 × 16	Bneck, 3 × 3	16	-	GELU	1
1122 × 16	Bneck, 3 × 3	24	-	GELU	2
562 × 24	Bneck, 3 × 3	24	-	GELU	1
562 × 24	ICA-Bneck, 5 × 5	40	√	GELU	2
282 × 40	ICA-Bneck, 5 × 5	40	√	GELU	1
282 × 40	ICA-Bneck, 5 × 5	40	√	GELU	1
282 × 40	Bneck, 3 × 3	80	-	Hard-swish	2
142 × 80	Bneck, 3 × 3	80	-	Hard-swish	1
142 × 80	Bneck, 3 × 3	80	-	Hard-swish	1
142 × 80	Bneck, 3 × 3	80	-	Hard-swish	1
142 × 80	ICA-Bneck, 3 × 3	112	√	Hard-swish	1
142 × 112	ICA-Bneck, 3 × 3	112	√	Hard-swish	1
142 × 112	ICA-Bneck, 5 × 5	160	√	Hard-swish	2
72 × 160	ICA-Bneck, 5 × 5	160	√	Hard-swish	1
72 × 160	ICA-Bneck, 5 × 5	160	√	Hard-swish	1
72 × 160	Conv2d, 1 × 1	960	-	Hard-swish	1
72 × 960	Pool, 7 × 7	-	-	-	1
12 × 960	Cond2d, 1 × 1, NBN	1280	-	Hard-swish	1
12 × 1280	Cond2d, 1 × 1, NBN	6	-	-	1

**Table 3:** Impact of different activation functions on model performance.

Activation Function	Accuracy (%)	Precision (%)	Recall (%)	F1-Score (%)
ReLU	91.96	91.94	91.92	91.92
LeakyReLU	92.08	92.22	92.07	92.07
ReLU6	92.08	92.14	92.04	92.04
GELU	92.32	92.43	92.29	92.28

**Table 4:** Impact of different attention mechanisms on model performance.

Attention Mechanism	Accuracy (%)	Precision (%)	Recall (%)	F1-Score (%)
SE	92.32	92.43	92.29	92.28
CBAM	91.02	91.02	90.95	90.95
ECA	92.79	92.83	92.76	92.77
CA	93.03	93.06	92.98	92.97
ICA	93.38	93.38	93.32	93.33

**Table 5:** Ablation experiment results of the T-GSR model.

Model	Image Preprocessing	Adaptive Residual Branch	GELU	ICA	Accuracy (%)	F1-Score (%)	Parameters (M)	FLOPs (G)
MobileNet V3	×	×	×	×	89.95	89.89	4.210	0.233
	√	×	×	×	91.02	90.92	4.210	0.233
	√	×	√	×	91.96	91.91	4.210	0.233
	√	×	×	√	92.08	92.07	2.993	0.236
	√	√	×	×	91.96	91.92	4.242	0.239
	√	√	√	×	92.32	92.28	4.242	0.239
T-GSR	√	√	√	√	93.38	93.33	3.025	0.242

**Table 6:** Comprehensive performance comparison of models.

Model	Accuracy (%)	Precision (%)	Recall (%)	F1-Score (%)	Parameters (M)	FLOPs (G)
ResNet18	90.19	90.24	90.11	90.11	11.180	1.824
RegNet	91.49	91.53	91.44	91.45	5.670	0.616
ShuffleNet V2	87.94	88.17	87.88	87.90	5.357	0.596
T-GSR	93.38	93.38	93.32	93.33	3.025	0.242

## Data Availability

The raw data supporting the conclusions of this article will be made available by the authors on request.
